# Impaired tumor angiogenesis and VEGF-induced pathway in endothelial CD146 knockout mice

**DOI:** 10.1007/s13238-014-0047-y

**Published:** 2014-04-24

**Authors:** Qiqun Zeng, Zhenzhen Wu, Hongxia Duan, Xuan Jiang, Tao Tu, Di Lu, Yongting Luo, Ping Wang, Lina Song, Jing Feng, Dongling Yang, Xiyun Yan

**Affiliations:** 1 Key Laboratory of Protein and Peptide Pharmaceuticals, CAS-University of Tokyo Joint Laboratory of Structural Virology and Immunology, Institute of Biophysics, Chinese Academy of Sciences, Beijing, 100101 China; 2 University of Chinese Academy of Sciences, Beijing, 100049 China; 3 Cardiovascular Research Institute, University of California, San Francisco, 555 Mission Bay Blvd. South, San Francisco, CA 94158 USA

**Keywords:** CD146, tumor angiogenesis, VEGF, knockout mice

## Abstract

CD146 is a newly identified endothelial biomarker that has been implicated in angiogenesis. Though *in vitro* angiogenic function of CD146 has been extensively reported, *in vivo* evidence is still lacking. To address this issue, we generated endothelial-specific CD146 knockout (CD146^EC-KO^) mice using the Tg(Tek-cre) system. Surprisingly, these mice did not exhibit any apparent morphological defects in the development of normal retinal vasculature. To evaluate the role of CD146 in pathological angiogenesis, a xenograft tumor model was used. We found that both tumor volume and vascular density were significantly lower in CD146^EC-KO^ mice when compared to WT littermates. Additionally, the ability for sprouting, migration and tube formation in response to VEGF treatment was impaired in endothelial cells (ECs) of CD146^EC-KO^ mice. Mechanistic studies further confirmed that VEGF-induced VEGFR-2 phosphorylation and AKT/p38 MAPKs/NF-κB activation were inhibited in these CD146-null ECs, which might present the underlying cause for the observed inhibition of tumor angiogenesis in CD146^EC-KO^ mice. These results suggest that CD146 plays a redundant role in physiological angiogenic processes, but becomes essential during pathological angiogenesis as observed in tumorigenesis.

**Electronic supplementary material:**

The online version of this article (doi:10.1007/s13238-014-0047-y) contains supplementary material, which is available to authorized users.

## **INTRODUCTION**

The vascular system develops and matures through two fundamental processes, vasculogenesis and angiogenesis. Vasculogenesis is restricted to embryogenesis, and represents the formation of primary capillary plexus from endothelial progenitor cells. Angiogenesis on the other hand, occurs in both embryo and adult, and refers to the formation of new blood vessels from the pre-existing vasculature under either physiologically or pathologically condition (Flamme et al., 1997). Examples of physiological angiogenesis include the formation of new blood vessels in the process of development, wound healing and the adult female reproductive cycle; and it is tightly balanced between pro-angiogenic and anti-angiogenic signals. In contrast, pathological angiogenesis is observed in a wide range of human diseases, including tumor growth, diabetic retinopathy and chronic inflammation. Different from physiological angiogenesis, pathological angiogenesis results from excessive pro-angiogenic signals and a lack of sufficient factors to mediate vessel maturation (Chung et al., 2010).

The regulation of angiogenesis is heavily influenced by a number of growth factors, among which VEGF has been identified as one of the most prominent factors, acting mainly through its endothelial receptor VEGFR2 (also known as Flk-1; Carmeliet and Jain, 2011). Targeted deletion of VEGFR-2 or VEGF in mice results in a complete lack of vascular development and leads to early embryonic lethality, demonstrating that the VEGF/VEGFR-2 pathway is essential for angiogenesis (Shalaby et al., 1995; Carmeliet et al., 1996). The binding of VEGF to VEGFR2 induces receptor dimerization and phosphorylation, which in turn triggers downstream signaling cascades including phosphorylation of p38, ERK1/2 MAPK and AKT, to promote endothelial cells (ECs) migration, proliferation and survival (Zachary and Gliki, 2001; Ferrara et al., 2003).

Another group of cell surface molecules, namely cell adhesion molecules, are essential for mediating cell-cell interactions, and play an essential role in the process of angiogenesis (Telo et al., 1997; Petruzzelli et al., 1999). Amongst them, CD146, also known as MCAM or Muc18, is a member of the immunoglobulin superfamily, which was originally identified as a marker for malignant melanoma (Lehmann et al., 1989; Xie et al., 1997). Subsequent studies revealed that CD146 is highly expressed in the endothelium (Solovey et al., 2001), and serves as a structural component of endothelial junctions (Bardin et al., 2001). Also, the finding that an anti-CD146 antibody, AA98, inhibits endothelial cell migration and tube formation *in vitro* and tumor angiogenesis in mice, established the important role of CD146 in angiogenesis (Yan et al., 2003). Recently, CD146 was identified as a co-receptor for VEGFR-2 to mediate the VEGF/VEGFR2 pathway (Jiang et al., 2012). To date, however, due to the lack of a CD146 conditional knockout mouse, most studies on the role of CD146 in angiogenesis are *in vitro* assays on cultured cell lines; *in vivo* studies are limited to zebrafish (Chan et al., 2005; So et al., 2010) and xenograft tumor models.

To gain a better understanding of the angiogenic functions of CD146 *in vivo*, we generated endothelial CD146 knockout (CD146^EC-KO^) mice using the Tg(Tek-cre) system. *In vitro* and *in vivo* angiogenesis studies were conducted on these mice. When compared to wild type (WT) littermates, *in vivo* tumor growth and angiogenesis were found to be significantly inhibited in CD146^EC-KO^ mice. We also found that ECs isolated from CD146^EC-KO^ mice were impaired in their ability for spouting, migration and tube formation in response to VEGF treatment. Importantly, the VEGF-induced VEGFR-2 phosphorylation and AKT/p38 MAPKs/NF-κB activation was found to be significantly inhibited in these CD146-null ECs. In conclusion, our results provide new insights into the mechanisms of pathological angiogenesis, and further confirmed our previous finding that CD146 plays an important role in VEGF/VEGFR2 pathway in the process of tumor angiogenesis.

## **RESULTS**

### **Generation of endothelial CD146 knockout mice**

**Figure 1:**
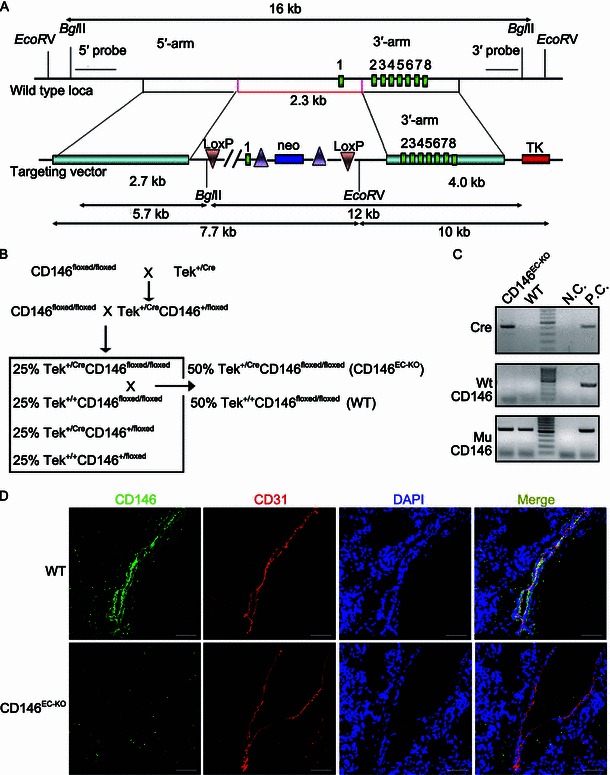
**Generation of endothelial-specific CD146 knockout mice**. (A) Targeting strategy for generation of *CD146*^*floxed*/*floxed*^ mice, shown are the wild type locus of mouse *CD146* gene (top), and the targeting construct (bottom). A LoxP site (3′loxp) was cloned upstream of the promoter, and the frt-Neo-frt-loxp cassette was cloned downstream of exon 1. (B) Mating scheme to generate endothelial-specific CD146 knock-out mice (*Tek*^*Cre*/+^*CD146*^*floxed*/*floxed*^, namely CD146^EC-KO^) and control WT littermates (*Tek*^+/+^*CD146*^*floxed*/*floxed*^). (C) Genotyping of CD146^EC-KO^ and WT mice by PCR analysis of genomic DNA. A 481-bp fragment from wild-type *Cre* gene, a 418-bp fragment from wild-type *CD146* gene (wt CD146) and a 537-bp fragment from floxed *CD146* gene (Mu CD146) were PCR-amplified with specific primers. Genomic DNA from Tg(Tek-Cre) mice was used as positive control (P.C.) for *Cre* analysis; Genomic DNA from *CD146*^*floxed*/*floxed*^ mice were used as P.C. for Mu CD146 analysis; genomic DNA from C57BL/6 mice were used as P.C. for wt CD146 analysis. ddH_2_O was used as negative control (N.C.) for all three PCR analyses. (D) Double immunofluorescence staining of CD31 and CD146 in lung tissues from WT and CD146^EC-KO^ mice. Scale bar, 50 μm

Mapping and nucleotide sequence analysis verified that the retrieved DNA sequence contained the promoter region and the initiating methionine of the murine CD146 gene, corresponding to the published CD146 cDNA sequence (Kohama et al., 2005). To generate CD146 conditional knockout mice (*CD146*^*floxed*/*floxed*^ mice), the promoter and 1^st^ exon of the CD146 gene were flanked with two inverted loxP sites, by cloning a LoxP site (3′loxp) upstream of the promoter, and a frt-Neo-frt-loxp cassette was cloned downstream of exon 1 (Fig. 1A). To further delete CD146 in ECs, we employed two mouse strains, *CD146*^*floxed*/*floxed*^ mice and *Tek*^+/*Cre*^ mice, in which the Cre gene was introduced into one allele of the Tek locus and is specifically expressed in ECs. To generate endothelial-specific CD146 knockout mice (CD146^EC-KO^ mice), we first crossed *CD146*^*floxed*/*floxed*^ with *Tek*^+/*Cre*^ mice. The resulting *Tek*^+/*Cre*^*CD146*^+/*floxed*^ mice were subsequently mated with *CD146*^*floxed*/*floxed*^ mice to generate *Tek*^+/*Cre*^*CD146*^*floxed*/*floxed*^ mice (Fig. 1B). The expected ratio of obtaining *Tek*^+/*Cre*^*CD146*^*floxed*/*floxed*^, *Tek*^+/*Cre*^*CD146*^+/*floxed*^, *Tek*^+/+^*CD146*^*floxed*/*floxed*^, *Tek*^+/+^*CD146*^+/*floxed*^ mice was 1:1:1:1. As *Tek*^+/*Cre*^*CD146*^*floxed*/*floxe*^ mice (CD146^EC-KO^ mice) were viable, these mice were further bred to *Tek*^+/+^*CD146*^*floxed*/*floxed*^ mice (WT mice), resulting in 50 % CD146^EC-KO^ mice and 50 % WT mice, both of which were used for subsequent investigations (Fig. 1B). Genomic DNA was isolated to verify the expected genotypes by PCR (Fig. 1C).

To demonstrate that the CD146 gene was inactivated in an endothelial-specific manner, lung tissues of CD146^EC-KO^ mice were prepared and analyzed by immunofluorescence using anti-CD146 and anti-CD31 antibodies. As shown in Fig. 1D, WT mice expressed the largest amount of CD146 in lung ECs as identified by CD31-positive staining. In contrast, CD146 expression was especially deficient in lung ECs of CD146^EC-KO^ mice. We also observed the absence of CD146 in ECs of kidney and liver via immunohistochemistry in CD146^EC-KO^ mice (Fig. S1). Despite endothelial deletion of CD146, CD146^EC-KO^ mice did not exhibit overt defects or detectable abnormalities in organ morphology upon analysis by light microscopy (data not shown).

### **Normal development of retinal vasculature in CD146**^**EC-KO**^**mice**

**Figure 2:**
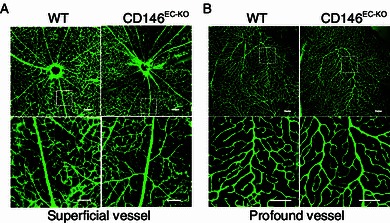
**Normal development of retinal vasculature in CD146**^**EC-KO**^**mice**. Fluorescein isothiocyanate-dextran-perfused retinas isolated from WT and CD146^EC-KO^ mice, superficial radial vessels (A) and profound vessels (B). Scale bar, 100 μm. *n* = 3 mice in each group

Since the retinal vasculature is an excellent model system to study the general development of blood vessels (Gariano and Gardner, 2005), we performed fluorescein angiography, to examine the vascular network in CD146^EC-KO^ mice. As shown in Fig. 2, there were no apparent differences in blood vessel density between CD146^EC-KO^ mice and their WT littermates. Our data revealed that large vessels sprouting from the optic nerve head (Fig. 2A) and small branching vessels (Fig. 2B) were also similar between the two groups. No abnormalities in retinal vasculature structure (including tortuosity, vessel dilatation and hemorrhages) in CD146^EC-KO^ mice were observed. Similarly, there were no differences in the morphology or density of skin vessel between WT and CD146^EC-KO^ mice (data not shown). These results suggest that endothelial deletion of CD146 does not affect normal retinal vascular development.

### **Impaired tumor growth in CD146**^**EC-KO**^**mice**

**Figure 3:**
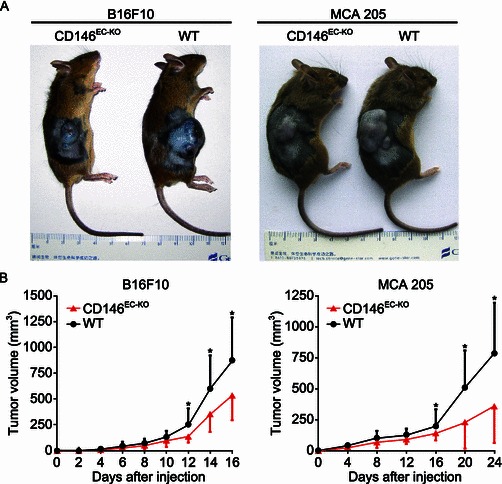
**Impaired tumor growth in CD146**^**EC-KO**^**mice**. B16F10 melanoma or MCA 205 fibrosarcoma were injected into CD146^EC-KO^ and WT mice. Tumor volume was monitored every 48 h. (A) Representative tumors in CD146^EC-KO^ and WT mice on the day when all the animals were sacrificed (16th day for B16F10 and 24th day for MCA 205 after injection). (B) Growth curve of tumors in CD146^EC-KO^ and WT mice. (*, *P* < 0.05 versus WT, *n* = 9 mice for melanoma injection, *n* = 10 mice for fibrosarcoma injection in each group)

To investigate the role of CD146 in pathological angiogenesis *in vivo*, the tumor growth in WT and CD146^EC-KO^ mice was measured, following subcutaneous injection of either a mouse melanoma cell line B16F10, or a fibrosarcoma cell line MCA 205, both of which are malignant tumors characterized by intense angiogenesis. Representative images of tumors in each group of mice at day 16 or day 24 after injection are shown in Fig. 3A. Solid tumors, formed after injection of cells from either tumor cell line, were both smaller in CD146^EC-KO^ mice compared to those in WT mice. When the excised tumors were analyzed, the B16F10 tumor size was nearly 40% smaller in CD146^EC-KO^ mice than WT mice (*P* < 0.05). The MCA 205 tumor size was almost 50% smaller in CD146^EC-KO^ mice than WT mice (*P* < 0.05) (Fig. 3B). These data suggest that endothelial deletion in mice of CD146 results in inhibition of tumor growth.

### **Impairment of tumor angiogenesis in CD146**^**EC-KO**^**mice**

**Figure 4:**
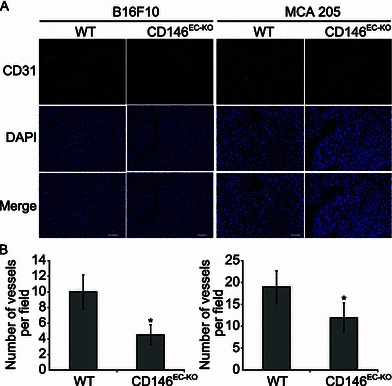
**Decreased tumor angiogenesis in CD146**^**EC-KO**^**mice**. (A) Representative microvessels of tumor sections in WT and CD146^EC-KO^ mice. Microvascular morphology as identified by fluorescence microscopy of tumor sections stained with an anti-CD31 antibody (red) and counterstained with DAPI (blue). Scale bar, 100 μm. (B) Microvessel counts in B16F10 and MCA 205 tumor sections from WT and CD146^EC-KO^ mice. The graph represents the average vessel number in three random (100×) fields of tumor section. Each tumor section was taken from the tumor edge of each mice. *n* = 6 mice in each group. (*, *P* < 0.05 versus WT)

To investigate whether impaired tumor growth in CD146^EC-KO^ mice was a consequence of impaired host angiogenesis, we compared tumor vessel density in tumor sections from CD146^EC-KO^ mice and WT mice. Immunofluorescence analysis with antibody targeting endothelial cell marker CD31 revealed that tumor sections in CD146^EC-KO^ mice displayed decreased vascular density compared with those in WT mice (Fig. 4A). The endothelial-specific deletion of CD146 in CD146^EC-KO^ mice was further confirmed in tumor sections (Fig. S2). Furthermore, to quantitate the overall degree of vascular density in tumor sections, vessels with CD31-positive staining were counted. As shown in Fig. 4B, vascular density in the CD146^EC-KO^ mice was significantly decreased in B16F10 tumors (9.9 ± 2.2 versus 4.5 ± 1.3 microvessels per field, *P* < 0.05) and MCA 205 tumors (18.7 ± 3.8 versus 11.6 ± 3.3 microvessels per field, *P* < 0.05). Thus, impaired tumor growth correlated with decreased vascular formation in CD146^EC-KO^ mice, suggesting that decreased vascular formation resulted in the impaired tumor growth in CD146^EC-KO^ mice.

### **Reduced VEGF-induced ECs sprouting in aortic ring of CD146**^**EC-KO**^**mice**

**Figure 5:**
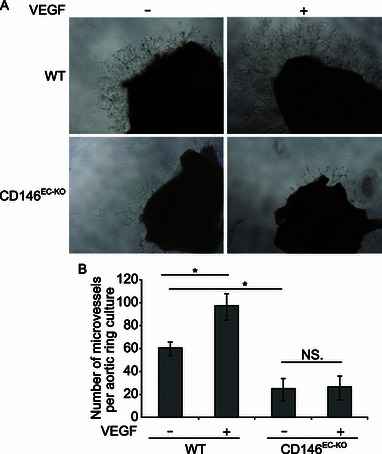
**Reduction in VEGF-induced EC sprouting in aortic rings of CD146**^**EC-KO**^**mice**. (A) Representative micrographs of WT and CD146^EC-KO^ aortic ring microvessels without or with VEGF (50 ng/mL) treatment. (B) Microvessel numbers were counted from WT and CD146^EC-KO^ aortic rings without or with VEGF (50 ng/mL) treatment. *n* = 3 mice per genotype; *, *P* < 0.05, NS., no significant differences, *P* > 0.05

To investigate further how angiogenesis functions in CD146^EC-KO^ mice, we performed an aortic ring assay. Since VEGF is the most prominent factor amongst the angiogenic factors many tumors secrete to promote new blood vessel formation (Grothey and Galanis, 2009), it was used as a stimulatory ligand in this model. As shown in Fig. 5, the aortic ring cultures isolated from CD146^EC-KO^ mice exhibited significantly reduced endothelial cell sprouting when compared to WT samples (*P* < 0.05). More interestingly, while the number of microvessels sprouting from the WT aortic ring cultures increased significantly in response to VEGF treatment, the number of microvessels sprouting in CD146^EC-KO^ mice remained unchanged. Together, these data support a critical role for CD146 in endothelial function and in angiogenesis, and suggest that VEGF-induced endothelial cell sprouting is inhibited in the absence of CD146.

### **Reduced VEGF-induced migration and tube formation in ECs of CD146**^**EC-KO**^**mice**

**Figure 6:**
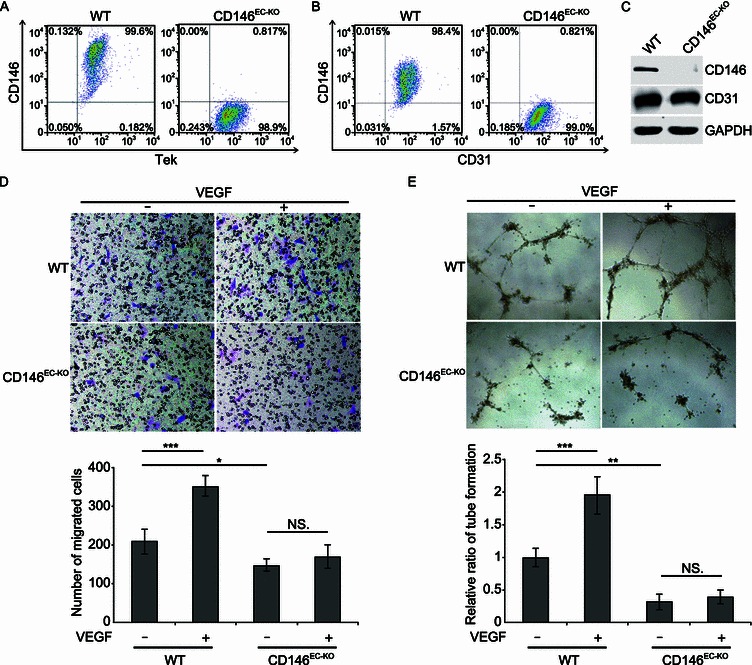
**Reduced VEGF-induced migration and tube formation in CD146-null ECs**. (A) FACS analysis of Tek and CD146 expression in ECs isolated from WT and CD146^EC-KO^ mice. (B) FACS analysis of CD31 and CD146 expression in ECs isolated from WT and CD146^EC-KO^ mice. (C) Western blot analysis of CD31 and CD146 expression in ECs isolated from WT and CD146^EC-KO^ mice. GAPDH were used as control. (D) Migration assay of ECs isolated from WT and CD146^EC-KO^ mice without or with VEGF (50 ng/mL) treatment. (E) Tube formation assay of ECs isolated from WT and CD146^EC-KO^ mice without or with VEGF (50 ng/mL) treatment. *, *P* < 0.05, NS., no significant differences, *P* > 0.05

The results from the *in vivo* tumor model and *in vitro* aortic ring model indicate that the loss of endothelial CD146 function leads to an inhibition of tumor angiogenesis. To further investigate this, we isolated liver ECs from WT and CD146^EC-KO^ mice and compared their capabilities of migration and tube formation *in vitro*. We first observed that ECs isolated from these two groups of mice showed no differences in morphology (Fig. S3). Cells were also verified to be of endothelial identity by FACS analysis and Western blot, as shown in Fig. 6A–C. While ECs isolated from WT mice displayed a CD31^+^/CD146^+^ or Tek^+^/CD146^+^ double positive phenotype, ECs from CD146^EC-KO^ mice showed a CD31^+^/CD146^-^ or Tek^+^/CD146^-^ single positive phenotype, verifying complete endothelial deletion of CD146 in these mice. In addition, mRNA level of other adhesion molecules, including JAM, PECAM-1, ICAM and VCAM remained unchanged (Fig. S4). Furthermore, we found that significantly fewer single cells migrated through the filter, and less of the tube-like network was formed on Matrigel for CD146-null ECs when compared with wild-type ECs. Importantly, the ability of CD146-null ECs for migration and tube formation was also significantly impaired in response to VEGF (Fig. 6D and 6E), suggesting that VEGF-induced endothelial activation is dependent on the presence of CD146. These observations were consistent with our previous finding that the disruption of CD146 function via targeting antibodies or siRNAs inhibits VEGF-induced cell migration and tube formation in human umbilical vein endothelial cells, HUVECs (Jiang et al., 2012).

### **Inhibition of VEGF-mediated signal transduction in CD146-null ECs**

**Figure 7:**
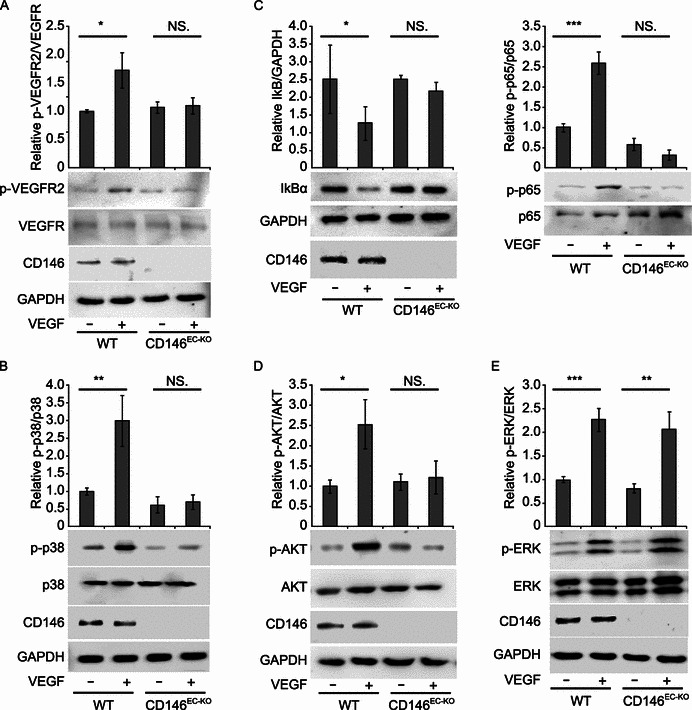
**Inhibition of VEGF-mediated signal transduction in CD146-null ECs**. (A) Phosphorylation of VEGFR-2 upon VEGF stimulation (50 ng/mL, 10 min) was determined in ECs from WT and CD146^EC-KO^ mice. (B) Activation of p38 induced by VEGF (50 ng/mL, 30 min) was measured in ECs from WT and CD146^EC-KO^ mice. (C) Degradation of I-κB and activation of NF-κB p65 induced by VEGF (50 ng/mL, 7 h) were determined in ECs from WT and CD146^EC-KO^ mice. (D and E) AKT and ERK activation induced by VEGF (50 ng/mL, 30 min) were measured in ECs isolated from WT and CD146^EC-KO^ mice. All Western blots were quantified by measuring the band density. Bar graphs (mean ± SD) present normalized values from at least 3 independent experiments. ***, *P* < 0.001, **, *P* < 0.01, *, *P* < 0.05, NS., no significant differences, *P* > 0.05

Mounting evidences indicate that there is a functional relationship between CD146 and VEGF (Jiang et al., 2012), we therefore focused on investigating whether the VEGF/VEGFR-2 signaling pathway was compromised in CD146-null ECs. As shown in Fig. 7, we observed that in wild-type ECs, VEGF-induced VEGFR-2 phosphorylation normally, as well as the p38/IKK/NF-κB signaling cascade, and Akt phosphorylation. In contrast, VEGF-induced activation signaling was significantly abrogated in CD146-null ECs. Interestingly, VEGF-induced ERK activation was not affected by the absence of CD146 (Fig. 7E). These data suggest that CD146 may play an important role in the Akt and p38/IKK/NF-κB pathways induced by VEGF, whilst other VEGF pathways appear to function in a CD146-independent manner. These observations were consistent with our previous finding that disruption of CD146 inhibits VEGF pathway in HUVECs (Jiang et al., 2012), providing an important clue for elucidating the precise molecular mechanisms responsible for the impairment of endothelial function, as well as disruption in tumor angiogenesis observed in CD146^EC-KO^ mice.

## **DISCUSSION**

In this report, endothelial-specific CD146 knockout mice (CD146^EC-KO^ mice) were generated via Cre/LoxP system and studies were performed on these mice to investigate in detail the involvement of CD146 during *in vivo* angiogenesis. These mice were viable, with no apparent morphological defects. However, CD146^EC-KO^ adult mice exhibited defective tumor angiogenesis, resulting in significantly delayed tumor growth. In confirmation, isolated ECs lacking CD146 performed poorly in spouting, migration and tube formation assays when compared to WT cells. When investigating the possible underlying mechanisms for the observed impairments, we found that VEGF-induced p38 signaling was greatly inhibited in ECs of CD146^EC-KO^ mice. Taken together, our data present here indicate the important role of endothelial CD146 in the process of *in vivo* blood vessel formation, and reveal that CD146 is critically involved in pathological angiogenesis via functional cooperation with the VEGF/VEGFR-2 pathway.

CD146 was previously found to be highly expressed in the endothelium (Shih, 1999), and subsequent studies established its role during *in vivo* angiogenesis, by finding that an anti-CD146 antibody, AA98, could inhibit tumor angiogenesis in xenografted mice (Yan et al., 2003). Generation of endothelial CD146 knockout mice here enabled us to systemically study the role of CD146 in angiogenesis *in vivo*. These endothelial-specific CD146-deficient mice exhibited normal development, suggesting that CD146 is dispensable for vasculogenesis in the process of embryogenesis; these animals had the ability to reproduce, which also suggests that CD146 does not play an essential role during physiological angiogenesis in adult mice, such as the adult female reproductive cycle. However, two previous studies established a role of CD146 in vascular development in zebrafish, by demonstrating that the suppression of CD146 affected vascular lumen formation of intersomitic vessels (Chan et al., 2005), and also angiogenic sprouting of intersegmental vessels (So et al., 2010), which is in apparent contradiction with our mouse studies. However, these two studies differ in two important ways from ours presented here. Firstly, their study on the role of CD146 in zebrafish was performed by way of anti-CD146 morpholino transient transfection, which abolished the expression of CD146 in all kinds of cells. In contrast, our targeted disruption of CD146 in mice was focused on Tek-positive cells, mainly including ECs and pericytes (Armulik et al., 2005). Secondly, embryogenesis is a more complex process in mice than that in zebrafish. A variety of endothelial cell adhesion molecules share certain functions with CD146 in angiogenic processes, such as JAM (Dejana et al., 2001), PECAM-1 (Graesser et al., 2002; Gratzinger et al., 2003) and ESAM (Hirata et al., 2001). All of these adhesion molecules might play overlapping roles with CD146, and could thus be able to compensate for its deletion *in vivo* during mouse embryogenesis, resulting in normal physiological angiogenesis in CD146^EC-KO^ mice. In contrast, CD146^EC-KO^ adult mice exhibited defective tumor angiogenesis and delayed tumor growth, suggesting its role in pathological angiogenic processes. When investigating the possible underlying mechanisms, we found that the migration and tube formation activities of CD146 knockout ECs were impaired, which may have led to impaired ECs function in CD146^EC-KO^ mice resulted in defective tumor angiogenesis. Besides, there might be two other potential mechanisms. Firstly, as revealed by a recent study, endothelial CD146 plays an important role in lymphocytes infiltration (Duan et al., 2013), thus the altered lymphocytes infiltration and cytokines expression in the tumor environment of CD146^EC-KO^ mice might have affected tumor angiogenesis. Secondly, since CD146 is expressed on the pericytes (Li et al., 2003; Crisan et al., 2009) and has also been deleted in CD146^EC-KO^ mice (Data not shown), CD146-null pericytes might have contributed to defective tumor angiogenesis.

The exact signaling mechanisms underlying CD146 function in angiogenesis has been the subject of several studies. Anfosso et al. originally reported that the engagement of CD146 in HUVECs led to the association with tyrosine kinase FYN, followed by the phosphorylation of FAK and paxillin, suggesting that CD146 acts as a membrane receptor to participate in outside-in signaling (Anfosso et al., 1998; Anfosso et al., 2001). Our previous observations demonstrated that CD146 is essential for the activation of the p38/IKK/NF-κB signaling pathway in HUVECs (Bu et al., 2006; Zheng et al., 2009). A following study showed that VEGF mediates CD146 dimerization and downstream signaling in a NOX4-dependent manner (Zhuang et al., 2010), which aroused our interest on the association between CD146 and VEGF pathway and finally led to the important finding that CD146 is a co-receptor of VEGFR-2 in tumor angiogenesis. The data we presented here is a strong confirmation of this finding, revealing that CD146 enhances pathological tumor angiogenesis through mediating VEGF pathway. One interesting phenomenon we observed that VEGF-induced p38 and AKT activations were significantly inhibited in isolated ECs lacking CD146, while ERK activation was not affected. It has been reported that intracellular propagations of different VEGFR2 signaling translate into diverse endothelial functions (Koch et al., 2011). For instance, the phosphorylated tyrosine pY1214 of VEGFR2 allows recruitment of NCK and FYN as well as eventually activation of p38 MAPK pathway, which is important for EC migration (Lamalice et al., 2006), while VEGF/VEGFR2-induced activation of the RAS/RAF/ERK/MAPK pathway is responsible for EC proliferation (Meadows et al., 2001; Zachary, 2001). Therefore, our observation gave clues about investigating a specific role for CD146 in VEGFR2 biology via the dissection out of specific VEGF-induced pathways in which it is involved. For example, our *in vitro* assay on isolated ECs showed impaired EC migration in the absence of CD146, providing strong evidences for the specific involvement of CD146 in EC migration and p38 MAPK pathway, and we hypothesized that FYN would be a linker between them. Meanwhile, the unaffected ERK pathway suggests that CD146 does not participate in ECs proliferation. Further investigations into the molecular details are warranted.

Together with findings from previous reports describing the effects of antibody inhibition of CD146, this study also raises the possibility of CD146 as a potential target for cancer therapy. Firstly, since CD146 has also been identified as a novel molecule for inducing epithelial-mesenchymal transition (EMT) in tumor progression (Imbert et al., 2012; Liu et al., 2012; Zeng et al., 2012), not only does the absence or inhibition of CD146 function block tumor growth and angiogenesis; it is also likely to suppress tumor metastasis. Secondly, anti-CD146 therapy is likely to be well tolerated, since endothelial deletion of CD146 in mice does not affect some forms of physiological angiogenesis, and deleterious effects have not been observed in mice following anti-CD146 antibody treatment (Yan et al., 2003; Jiang et al., 2012). Last but most importantly, anti-CD146 therapy could present a promising approach to combine with existing strategies targeting VEGF pathway to inhibit tumor angiogenesis. Since many cancers activate VEGF-A expression and the great importance of VEGF in neovascularization has been emphasized, strategies to inactivate VEGF/VEGFR signaling have led to significant suppression of tumor angiogenesis and tumor growth (Ferrara and Alitalo, 1999; Brekken et al., 2000), including the anti-VEGF-A mAb bevacizumab (Ferrara et al., 2004), as well as two small molecule tyrosine kinase inhibitors (TKIs) sorafenib and sunitinib (Chung et al., 2010). However, it has been reported recently that a small percentage of patients acquired tolerance to these drugs, possibly due to the complexity of tumor angiogenic signaling (Van Cutsem et al., 2011). Our data reveal that the activation of VEGF/VEGFR is significantly impaired in the absence of CD146, suggesting that anti-VEGF and anti-CD146 adjunct therapies would have a cumulative effect on inhibiting tumor angiogenesis. Our observations described here and our previous studies have verified this hypothesis, and further studies should shed more light on the precise mechanisms involved in tumor angiogenesis.

## **MATERIALS AND METHODS**

### **Antibodies and reagents**

The rabbit anti-CD146 polyclonal antibody and mouse anti-CD146 monoclonal antibody AA1 and AA4 were generated in our laboratory (Zhang et al., 2008). Rat anti-mouse CD146 (clone ME-9F1) was purchased from BD Biosciences. Anti-mouse Tek-PE and anti-mouse CD31-APC antibodies were purchased from Tianjin Sungene Biotech Co., Ltd. Antibodies specific for phospho-p38 MAPK, p38 MAPK, phospho-NF-κB p65 (Ser536), phospho-ERK, ERK, AKT, were purchased from Cell Signaling Technology. Antibodies specific for I-κB were from Beijing Zhong Shan-Golden Bridge Biological Technology CO., LTD. Antibodies against phosphor-VEGFR-2 (Tyr 1214) and phospho-AKT (Ser 473) were purchased from Signalway Antibody. Antibodies specific for CD31 and GAPDH were purchased from Abcam. HRP-conjugated goat anti-mouse or rabbit IgG were purchased from GE Healthcare. Enhanced chemiluminescence assay kits were purchased from Pierce. Biotin-conjugated secondary antibodies and HRP-conjugated streptavidin were purchased from Dianova. Goat serum and the DAB substrate system were purchased from Santa Cruz Biotechnology. DAPI was purchased from Roche. Fluorescein isothiocyanate-dextran was purchased from Sigma. Growth factor-reduced Matrigel and collagenase were purchased from BD Biosciences. VEGF-A was purchased from Upstate Biotechnology.

### **Generation of endothelial cell-specific CD146 knockout mice**

This study was approved by the Biomedical Research Ethics Committee of the Institute of Biophysics, Chinese Academy of Sciences (Beijing, China), and all animal experiments were performed in compliance with the guidelines for the care and use of laboratory animals of the institute.

The conditional CD146 knockout mice (*CD146*^*floxed*/*floxed*^ mice) were generated in Model Animal Research Center of Nanjing University. Briefly, a 9 kb mouse DNA containing the CD146 gene was cloned into the pL253 vector. A LoxP site (3′loxp) was cloned upstream of the promoter, and the frt-Neo-frt-loxp cassette was cloned downstream of exon 1 (Fig. 1A). The linearized targeting vector was transfected into 129S6/SvEvTac-derived W4 embryonic stem (ES) cells by electroporation. The correctly targeted embryonic stem cell clones were injected into C57Bl/6 blastocysts to generate chimeric animals. Five chimeric male mice were obtained and bred to C57Bl/6 females (Jackson Laboratories) to obtain heterozygous pups. Heterozygous mice (*CD146*^*floxed*/+^) were backcrossed to C57Bl/6 for at least 5 generations before homozygous animals (*CD146*^*floxed*/*floxed*^) were generated.

To further generate endothelial-specific CD146 knockout mice (CD146^EC-KO^ mice), *CD146*^*floxed*/*floxed*^ mice were further bred to *Tek*^+/*Cre*^ mice (Strain Name: B6.Cg-Tg(Tek-cre)12Flv/J, The Jackson Laboratory), which specifically expressed Cre recombinase in ECs. The Mating schematic is shown in Fig. 1B. Briefly, *CD146*^*floxed*/*floxed*^ mice crossed with *Tek*^+/*Cre*^ mice generate *Tek*^+/*Cre*^*CD146*^*floxed*/+^ mice. These mice were subsequently backcrossed with *CD146*^*floxed*/*floxed*^ mice to generate endothelial-specific CD146 knockout (*Tek*^+/*Cre*^*CD146*^*floxed*/*floxed*^) and control (*Tek*^+^/^+^*CD146*^*floxed*/*floxed*^) mice.

*Tek*^+/*Cre*^*CD146*^*floxed*/*floxed*^ mice (CD146^EC-KO^ mice) were viable, and these mice were further cross-bred with *Tek*^+/+^*CD146*^*floxed*/*floxed*^ mice, resulting in 50 % *Tek*^+/*Cre*^*CD146*^*floxed*/*floxed*^ mice (CD146^EC-KO^ mice) and 50 % *Tek*^+/+^*CD146*^*floxed*/*floxed*^ mice (WT mice). Genotyping of knockout animals was performed by PCR analysis with tail DNA according to a standard protocol (Fig. 1C).

### **Immunohistochemistry**

Paraffin-embedded tissue sections were deparaffinized and hydrated. Endogenous peroxidase activity was quenched by incubation for 30 min with 0.3% H_2_O_2_ in methanol at 37°C. After washing with PBS, the tissue sections were boiled for 30 min in 10 mmol/L citrate buffer (pH 6.0) at 100°C. Once cooled down to room temperature, tissue sections were blocked for 1 h with 5% normal goat serum in PBS at 37°C, and then incubated overnight with rat anti-mouse CD146 antibody (Clone 9F1) or CD31 antibody at 4°C. After three washes in PBS for 5 min, tissue sections were incubated with biotin-conjugated secondary antibodies (1:1000 diluted) at 37°C for 1 h. After washing in PBS for 3 times, tissue sections were then incubated with HRP-conjugated streptavidin (1:1000 diluted) at 37°C for 45 min. Freshly prepared DAB was added for color development. All tissue sections were counterstained with haematoxylin. Finally, the stained tissue sections were analyzed under an OLYMPUS BX51 microscope.

### **Retinal fluorescein angiogram**

Mice were anesthetized, followed by exposure of the thoracic cavity. 1 mL PBS containing 25 mg of fluorescein isothiocyanate-dextran dye was injected into the left ventricular of the mice. Subsequently, eyes were removed and fixed in 4% paraformaldehyde for 3 h. Finally, the retina was peeled off and mounted on a glass slide. Fluorescent micrographs were taken with a confocal laser scanning microscope (FV-1000, Olympus).

### ***In vivo*tumor model**

Two tumor cell lines B16F10 melanoma and MCA 205 fibrosarcoma were used as *in vivo* tumor growth models. CD146^EC-KO^ mice that were 2–3 months, and both sex- and age-matched WT mice were used in this study. 1 × 10^6^ tumor cells in 100 μL of PBS were injected subcutaneously into the mice. Every other day, tumor volume was measured with calipers and calculated based on the formula (length × width × height). When the tumor volume reached about 1,500 mm^3^, all the mice were sacrificed and tumor tissues were peeled off for further analysis.

### **Aortic ring assay**

Mouse aortic ring assays were performed essentially as described previously (Baker et al., 2012). 1-mm thoracic aortic rings were placed in 50 μL growth factor-reduced Matrigel, and then overlaid with 100 μL of Opti-MEM with or without VEGF (50 ng/mL). Microvessel outgrowth was visualized by an inverted microscope (Eclipse model TS100; Nikon) with a CCD color camera (Model KP-D20AU; Hitachi) and the number of vessels growing from each aortic ring was counted at day 7 using Image Pro Plus software.

### **Isolation of ECs from WT and CD146**^**EC-KO**^**mice**

Sex- and age-matched CD146^EC-KO^ and WT mice were anesthetized, followed by exposure of the abdominal cavity. 30 mL of PBS was injected via the hepatic portal vein to flush the blood cells in the liver. Subsequently, 20 mL of collagenase (100 μg/mL dissolved in D-hanks buffer) were injected. The livers were subsequently removed, cut into pieces and then incubated with 2 mL of collagenase at 37°C for 10 min. 5 mL of DMEM medium containing 2% FBS was added and gently agitated for a few seconds. The resulting tissue/cell suspension was filtered through a 100 μm strainer (REF 352360, BD Biosciences). The filtered cell suspension was centrifuged for 1 min at 300 rpm, the supernatant was then centrifuged for 5 min at 500 rpm. Subsequently, the supernatant was centrifuged for 7 min at 2000 rpm. After removal of the supernatant, the cell pellet was washed once with DMEM and then resuspended in 12 mL of complete DMEM and plated into a gelatin-coated 6-well plate. The following day, the medium was exchanged with fresh complete DMEM, and the cells were cultured for an additional 1–2 days.

### **FACS analysis**

Isolated ECs from WT and CD146^EC-KO^ mice were trypsinized, washed with PBS, and then incubated with PE-conjugated AA1 and APC-conjugated CD31 antibody or APC-conjugated AA1 and PE-conjugated Tek for 45 min at 4°C. Cells were then washed three times with PBS, before analysis using a Becton Dickinson FACS Calibur flow cytometer.

### **Western blot**

Cell lysates of isolated ECs from WT and CD146^EC-KO^ mice were run on a 10% SDS-polyacrylamide gel and then transferred to a nitrocellulose membrane. Subsequently, nitrocellulose membranes were blocked for 60 min with 5% non-fat milk in PBS at room temperature, and then incubated over night with the primary antibodies at 4°C, followed by incubation with goat anti-mouse or anti-rabbit IgG conjugated to HRP for 45 min at room temperature. Enhanced chemiluminescence (Pierce) was used to detect the presence of specific immunoreactive proteins. The bands were quantified by Quantity One software.

### **Cell migration assay**

ECs isolated from WT and CD146^EC-KO^ mice were trypsinized, washed and then resuspended in fresh serum-free DMEM medium and counted. 1.2 × 10^4^ cells were resuspended in serum-free medium into the upper chamber of each well (96-well inserts, 8 μm, Corning), and treated with or without VEGF (50 ng/mL). Lower chambers contained fresh medium containing 10% fetal bovine serum serving as chemoattractant. After overnight incubation at 37°C, cells at the upper surface of the membrane were removed using a swab, and cells at the lower surface of the membrane were fixed with 4% paraformaldehyde at room temperature for 15 min, and subsequently stained for 15 min at room temperature with Crystal Violet. Finally, to remove any unincorporated Crystal Violet, cells were washed with water. Pictures were taken using an OLYMPUS BX51 microscope. Cells migrating through the filter were counted using Image J software.

### **Tube formation**

96-well plates were coated with 50 μL growth factor-reduced Matrigel. The isolated ECs from WT and CD146^EC-KO^ mice were trypsinized, washed, resuspended in fresh serum-free DMEM medium and counted. 2 × 10^4^ cells were cultured on the Matrigel and treated with or without VEGF (50 ng/mL) and incubated overnight at 37°C. Tube formation was analyzed with an inverted microscope (Eclipse model TS100; Nikon) with a CCD color camera (Model KP-D20AU; Hitachi) and the tube length was measured using Image Pro Plus software.

### **Activation of VEGFR-2 signaling pathway**

Isolated ECs from WT and CD146^EC-KO^ mice were starved with DMEM medium for 24 h and then induced with VEGF (50 ng/mL) at 37°C for 10 min, 30 min or 7 h for analysis of the activation of VEGFR-2, p38/AKT/ERK and NF-KB, respectively. Cells were then washed with PBS, lysed in RIPA lysis buffer (150 mmol/L NaCl, 1 mmol/L EDTA, 50 mmol/L Tris, pH 8.0, 10% glycerol, 1% NP-40, 1 mmol/L phenylmethylsulfonyl fluoride (PMSF), and 25 μg/mL aprotinin), prior to analysis of activation of the relevant signaling pathways by Western blotting, as described above.

### **Statistical analysis**

All values are representative of experiments performed in triplicate. Quantitative Data are expressed as mean ± SD. Statistical differences were determined by unpaired Student’s *t* tests. The statistical differences of the tumor model were determined by paired Student’s *t* tests. The criterion for statistical significance was defined as *P* < 0.05.

## Electronic supplementary material

Below is the link to the electronic supplementary material.

Supplementary material 1 (DOCX 20,867 kb)

## References

[CR1] Anfosso F, Bardin N, Frances V, Vivier E, Camoin-Jau L, Sampol J, Dignat-George F (1998). Activation of human endothelial cells via S-endo-1 antigen (CD146) stimulates the tyrosine phosphorylation of focal adhesion kinase p125 (FAK). J Biol Chem.

[CR2] Anfosso F, Bardin N, Vivier E, Sabatier F, Sampol J, Dignat-George F (2001). Outside-in signaling pathway linked to CD146 engagement in human endothelial cells. J Biol Chem.

[CR3] Armulik A, Abramsson A, Betsholtz C (2005). Endothelial/pericyte interactions. Circ Res.

[CR4] Baker M, Robinson SD, Lechertier T, Barber PR, Tavora B, D’Amico G, Jones DT, Vojnovic B, Hodivala-Dilke K (2012). Use of the mouse aortic ring assay to study angiogenesis. Nat Protoc.

[CR5] Bardin N, Anfosso F, Masse JM, Cramer E, Sabatier F, Le Bivic A, Sampol J, Dignat-George F (2001). Identification of CD146 as a component of the endothelial junction involved in the control of cell–cell cohesion. Blood.

[CR6] Brekken RA, Overholser JP, Stastny VA, Waltenberger J, Minna JD, Thorpe PE (2000). Selective inhibition of vascular endothelial growth factor (VEGF) receptor 2 (KDR/Flk-1) activity by a monoclonal anti-VEGF antibody blocks tumor growth in mice. Cancer Res.

[CR7] Bu P, Gao L, Zhuang J, Feng J, Yang D, Yan X (2006). Anti-CD146 monoclonal antibody AA98 inhibits angiogenesis via suppression of nuclear factor-kappaB activation. Mol Cancer Ther.

[CR8] Carmeliet P, Jain RK (2011). Molecular mechanisms and clinical applications of angiogenesis. Nature.

[CR9] Carmeliet P, Ferreira V, Breier G, Pollefeyt S, Kieckens L, Gertsenstein M, Fahrig M, Vandenhoeck A, Harpal K, Eberhardt C (1996). Abnormal blood vessel development and lethality in embryos lacking a single VEGF allele. Nature.

[CR10] Chan B, Sinha S, Cho D, Ramchandran R, Sukhatme VP (2005). Critical roles of CD146 in zebrafish vascular development. Dev Dyn.

[CR11] Chung AS, Lee J, Ferrara N (2010). Targeting the tumour vasculature: insights from physiological angiogenesis. Nat Rev Cancer.

[CR12] Crisan M, Chen CW, Corselli M, Andriolo G, Lazzari L, Peault B (2009). Perivascular multipotent progenitor cells in human organs. Hematop Stem Cells VII.

[CR13] Dejana E, Spagnuolo R, Bazzoni G (2001). Interendothelial junctions and their role in the control of angiogenesis, vascular permeability and leukocyte transmigration. Thromb Haemost.

[CR14] Duan HX, Xing S, Luo YT, Feng LQ, Gramaglia I, Zhang Y, Lu D, Zeng QQ, Fan KL, Feng J et al (2013) Targeting endothelial CD146 attenuates neuroinflammation by limiting lymphocyte extravasation to the CNS. Sci Rep 3 10.1038/srep01687 PMC3629416 23595028

[CR15] Ferrara N, Alitalo K (1999). Clinical applications of angiogenic growth factors and their inhibitors. Nat Med.

[CR16] Ferrara N, Gerber HP, LeCouter J (2003). The biology of VEGF and its receptors. Nat Med.

[CR17] Ferrara N, Hillan KJ, Gerber HP, Novotny W (2004). Discovery and development of bevacizumab, an anti-VEGF antibody for treating cancer. Nat Rev Drug Discov.

[CR18] Flamme I, Frolich T, Risau W (1997). Molecular mechanisms of vasculogenesis and embryonic angiogenesis. J Cell Physiol.

[CR19] Gariano RF, Gardner TW (2005). Retinal angiogenesis in development and disease. Nature.

[CR20] Graesser D, Solowiej A, Bruckner M, Osterweil E, Juedes A, Davis S, Ruddle NH, Engelhardt B, Madri JA (2002). Altered vascular permeability and early onset of experimental autoimmune encephalomyelitis in PECAM-1-deficient mice. J Clin Investig.

[CR21] Gratzinger D, Barreuther M, Madri JA (2003). Platelet-endothelial cell adhesion molecule-1 modulates endothelial migration through its immunoreceptor tyrosine-based inhibitory motif. Biochem Biophys Res Commun.

[CR22] Grothey A, Galanis E (2009). Targeting angiogenesis: progress with anti-VEGF treatment with large molecules. Nat Rev Clin Oncol.

[CR23] Hirata K, Ishida T, Penta K, Rezaee M, Yang E, Wohlgemuth J, Quertermous T (2001). Cloning of an immunoglobulin family adhesion molecule selectively expressed by endothelial cells. J Biol Chem.

[CR24] Imbert AM, Garulli C, Choquet E, Koubi M, Aurrand-Lions M, Chabannon C (2012). CD146 expression in human breast cancer cell lines induces phenotypic and functional changes observed in Epithelial to Mesenchymal Transition. PLoS ONE.

[CR25] Jiang TX, Zhuang J, Duan HX, Luo YT, Zeng QQ, Fan KL, Yan HW, Lu D, Ye Z, Hao JF (2012). CD146 is a coreceptor for VEGFR-2 in tumor angiogenesis. Blood.

[CR26] Koch S, Tugues S, Li XJ, Gualandi L, Claesson-Welsh L (2011). Signal transduction by vascular endothelial growth factor receptors. Biochem J.

[CR27] Kohama K, Tsukamoto Y, Furuya M, Okamura K, Tanaka H, Miki N, Taira E (2005). Molecular cloning and analysis of the mouse gicerin gene. Neurochem Int.

[CR28] Lamalice L, Houle F, Huot J (2006). Phosphorylation of Tyr(1214) within VEGFR-2 triggers the recruitment of Nck and activation of Fyn leading to SAPK2/p38 activation and endothelial cell migration in response to VEGF. J Biol Chem.

[CR29] Lehmann JM, Riethmuller G, Johnson JP (1989). MUC18, a marker of tumor progression in human melanoma, shows sequence similarity to the neural cell adhesion molecules of the immunoglobulin superfamily. Proc Natl Acad Sci USA.

[CR30] Li Q, Yu Y, Bischoff J, Mulliken JB, Olsen BR (2003). Differential expression of CD146 in tissues and endothelial cells derived from infantile haemangioma and normal human skin. J Pathol.

[CR31] Liu WF, Ji SR, Sun JJ, Zhang Y, Liu ZY, Liang AB, Zeng HZ (2012). CD146 expression correlates with epithelial–mesenchymal transition markers and a poor prognosis in gastric cancer. Int J Mol Sci.

[CR32] Meadows KN, Bryant P, Pumiglia K (2001). Vascular endothelial growth factor induction of the angiogenic phenotype requires Ras activation. J Biol Chem.

[CR33] Petruzzelli L, Takami M, Humes HD (1999). Structure and function of cell adhesion molecules. Am J Med.

[CR34] Shalaby F, Rossant J, Yamaguchi TP, Gertsenstein M, Wu XF, Breitman ML, Schuh AC (1995). Failure of blood-island formation and vasculogenesis in Flk-1-deficient mice. Nature.

[CR35] Shih IM (1999). The role of CD146 (Mel-CAM) in biology and pathology. J Pathol.

[CR36] So JH, Hong SK, Kim HT, Jung SH, Lee MS, Choi JH, Bae YK, Kudoh T, Kim JH, Kim CH (2010). Gicerin/CD146 is involved in zebrafish cardiovascular development and tumor angiogenesis. Genes Cells.

[CR37] Solovey AN, Gui L, Chang L, Enenstein J, Browne PV, Hebbel RP (2001). Identification and functional assessment of endothelial P1H12. J Lab Clin Med.

[CR38] Telo P, Lostaglio S, Dejana E (1997). Structure of intercellular junctions in the endothelium. Therapie.

[CR39] Van Cutsem E, Lambrechts D, Prenen H, Jain RK, Carmeliet P (2011). Lessons from the adjuvant bevacizumab trial on colon cancer: what next?. J Clin Oncol.

[CR40] Xie S, Luca M, Huang S, Gutman M, Reich R, Johnson JP, Bar-Eli M (1997). Expression of MCAM/MUC18 by human melanoma cells leads to increased tumor growth and metastasis. Cancer Res.

[CR41] Yan XY, Lin Y, Yang DL, Shen Y, Yuan M, Zhang ZQ, Li PY, Xia HT, Li L, Luo DD (2003). A novel anti-CD146 monoclonal antibody, AA98, inhibits angiogenesis and tumor growth. Blood.

[CR42] Zachary I (2001). Signaling mechanisms mediating vascular protective actions of vascular endothelial growth factor. Am J Physiol Cell Physiol.

[CR43] Zachary I, Gliki G (2001). Signaling transduction mechanisms mediating biological actions of the vascular endothelial growth factor family. Cardiovasc Res.

[CR44] Zeng Q, Li W, Lu D, Wu Z, Duan H, Luo Y, Feng J, Yang D, Fu L, Yan X (2012). CD146, an epithelial-mesenchymal transition inducer, is associated with triple-negative breast cancer. Proc Natl Acad Sci USA.

[CR45] Zhang Y, Zheng C, Zhang J, Yang D, Feng J, Lu D, Yan X (2008). Generation and characterization of a panel of monoclonal antibodies against distinct epitopes of human CD146. Hybridoma (Larchmt).

[CR46] Zheng C, Qiu Y, Zeng Q, Zhang Y, Lu D, Yang D, Feng J, Yan X (2009). Endothelial CD146 is required for in vitro tumor-induced angiogenesis: the role of a disulfide bond in signaling and dimerization. Int J Biochem Cell Biol.

[CR47] Zhuang J, Jiang T, Lu D, Luo Y, Zheng C, Feng J, Yang D, Chen C, Yan X (2010). NADPH oxidase 4 mediates reactive oxygen species induction of CD146 dimerization in VEGF signal transduction. Free Radic Biol Med.

