# Supplementary material for: Impaired tumor angiogenesis and VEGF-induced pathway in endothelial CD146 knockout mice
Source: Protein Cell. 2014 Apr 24;5(6):445–56. doi: 10.1007/s13238-014-0047-y (PMC4026419; doi:10.1007/s13238-014-0047-y)
Supplement: Supplementary file 1 — Supplementary material 1 (DOCX 20,867 kb) [file 13238_2014_47_MOESM1_ESM.docx]

**Impaired tumor angiogenesis and VEGF-induced pathway in endothelial CD146 knockout mice**

**Running title:** **In vivo angiogenesis in endothelial CD146 knockout mice**

**Keywords:** CD146; tumor angiogenesis; VEGF; knockout mice

Qiqun Zeng ^1*^, Zhenzhen Wu ^1,2*^, Hongxia Duan ^1,2^, Xuan Jiang ^3^, Tao Tu ^1,2^, Di Lu ^1^, Yongting Luo ^1^, Ping Wang ^1,2^, Lina Song ^1^, Jing Feng ^1^, Dongling Yang ^1^, Xiyun Yan ^1^

^1^ Key Laboratory of Protein and Peptide Pharmaceuticals, CAS-University of Tokyo Joint Laboratory of Structural Virology and Immunology, Institute of Biophysics, Chinese Academy of Sciences, 15 Datun Road, Beijing 100101, China

^2^ University of Chinese Academy of Sciences, 19A Yuquan Road, Beijing 100049, China

^3^ Cardiovascular Research Institute, University of California, San Francisco, 555 Mission Bay Blvd. South, San Francisco, California, USA 94158

Corresponding author: Xiyun Yan, 15 Datun Road, Chaoyang District, Beijing 100101, China. Telephone: +86 10 6488 8583; Fax: +86 10 6488 8584; E-mail: [yanxy@ibp.ac.cn](mailto:yanxy@ibp.ac.cn).

* These authors contributed equally to this work.

**Figure S1** CD146 is specifically deleted in endothelial cells. Immunohistochemical assay of CD146 expression on liver (A) and kidney (B) endothelial cells of WT and CD146^EC-KO^ mice. Scale bar, 100 μm.

**Figure S2** CD146 is specifically deleted in tumor vessels. Double immunofluorescence staining with CD146 and CD31 in tumors formed by MCA 205 cells. Scale bar, 50 μm.

**Figure S3** Morphology of endothelial cells isolated from WT and CD146^EC-KO^ mice.

**Figure S4** Real-time PCR analysis of CD146 and related adhesion molecules in ECs isolated from WT and CD146^EC-KO^ mice. ***, P<0.001; NS., no significant differences.

**Figure S1**


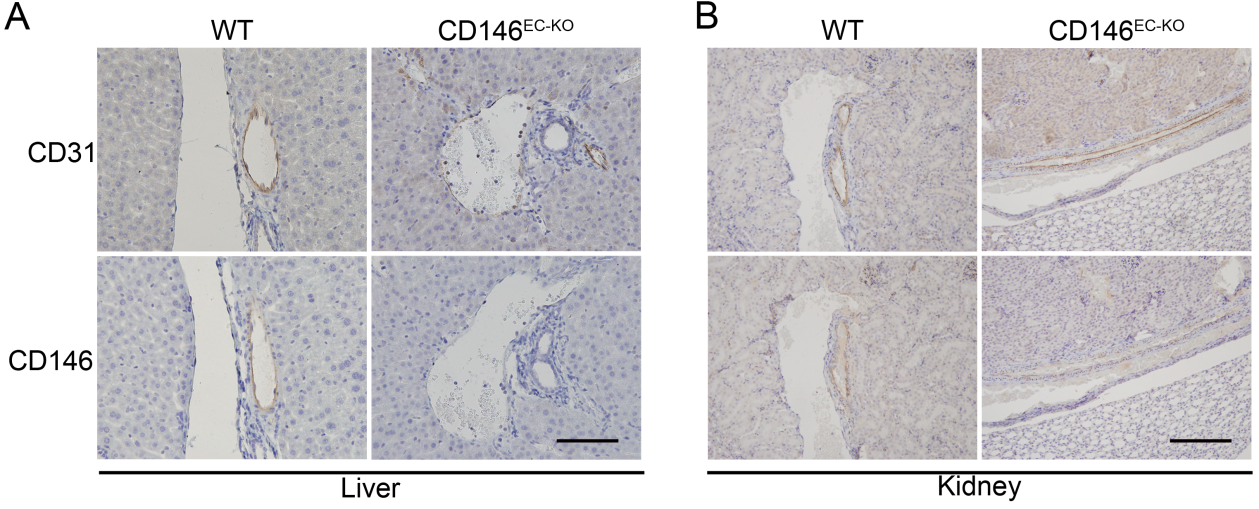


**Figure S2**


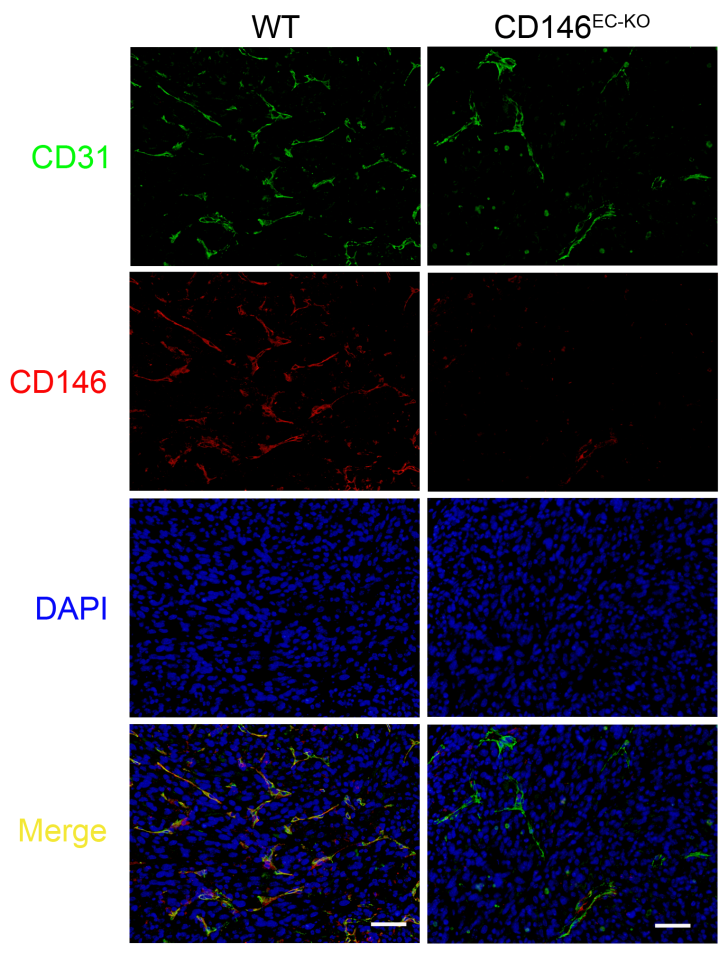


**Figure S3**


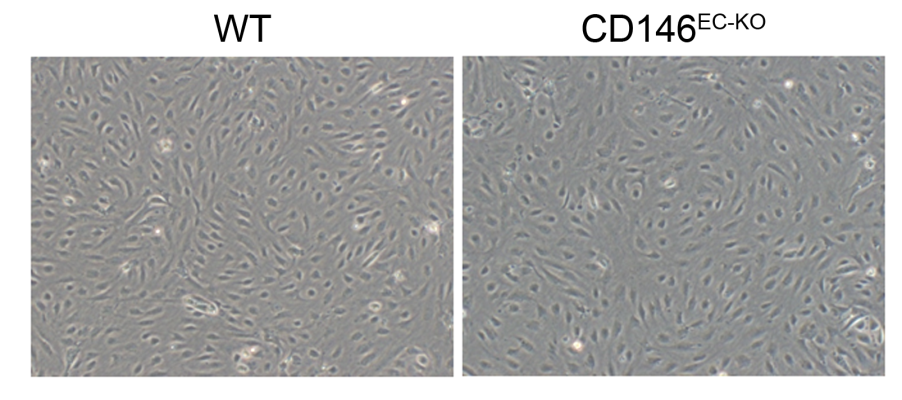


**Figure S4**

**
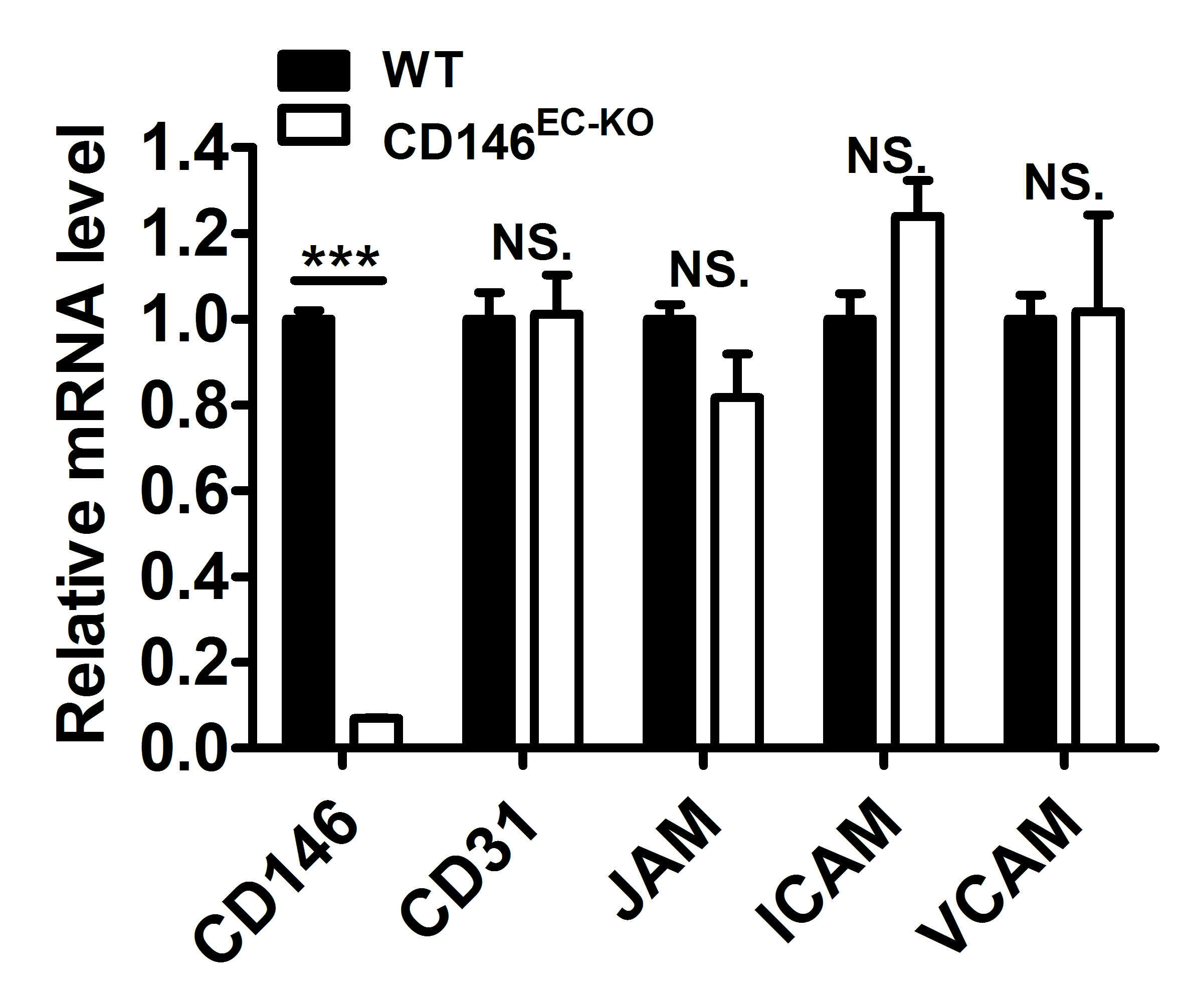
**
